# Accuracy and feasibility of point-of-care and continuous blood glucose analysis in critically ill ICU patients

**DOI:** 10.1186/cc5048

**Published:** 2006-09-18

**Authors:** Anouk M Corstjens, Jack JM Ligtenberg, Iwan CC van der Horst, Rob Spanjersberg, Joline SW Lind, Jaap E Tulleken, John HJM Meertens, Jan G Zijlstra

**Affiliations:** 1 Department of Anesthesiology, University Medical Center Groningen, P.O. Box 30.001, NL-9700 RB, Groningen, The Netherlands; 2 Intensive & Respiratory Care Unit (ICB), University Medical Center Groningen, P.O. Box 30.001, NL-9700 RB, Groningen, The Netherlands; 3 Department of Cardiology, University Medical Center Groningen, P.O. Box 30.001, NL-9700 RB Groningen, The Netherlands

## Abstract

**Introduction:**

To obtain strict glucose regulation, an accurate and feasible bedside glucometry method is essential. We evaluated three different types of point-of-care glucometry in seriously ill intensive care unit (ICU) patients. The study was performed as a single-centre, prospective, observational study in a 12-bed medical ICU of a university hospital.

**Methods:**

Patients with an expected ICU stay of more than 48 hours were included. Because the reference laboratory delivers glucose values after approximately 30 to 60 minutes, which is too slow to use in a glucose regulation protocol and for calibration of the subcutaneous continuous glucose monitoring system (CGMS) (CGMS System Gold), we first validated the ICU-based blood gas/glucose analyser ABL715 (part 1 of the study). Subsequently, part 2 was performed: after inserting (and calibrating) the subcutaneous CGMS, heparinised arterial blood samples were drawn from an arterial line every 6 hours and analysed on both the Precision PCx point-of-care meter using test strips and on the blood gas/glucose analyser ABL715. CGMS glucose data were downloaded after 24 to 72 hours. The results of the paired measurements were analysed as a scatter plot by the method of Bland and Altman and were expressed as a correlation coefficient.

**Results:**

Part 1: Four hundred and twenty-four blood samples were drawn from 45 critically ill ICU patients. The ICU-based blood gas/glucose analyser ABL715 provided a good estimate of conventional laboratory glucose assessment: the correlation coefficient was 0.95. In the Clarke error grid, 96.8% of the paired measurements were in the clinically acceptable zones A and B. Part 2: One hundred sixty-five paired samples were drawn from 19 ICU patients. The Precision PCx point-of-care meter showed a correlation coefficient of 0.89. Ninety-eight point seven percent of measurements were within zones A and B. The correlation coefficient for the subcutaneous CGMS System Gold was 0.89. One hundred percent of measurements were within zones A and B.

**Conclusion:**

The ICU-based blood glucose analyser ABL715 is a rapid and accurate alternative for laboratory glucose determination and can serve as a standard for ICU blood glucose measurements. The Precision PCx is a good alternative, but feasibility may be limited because of the blood sample handling. The subcutaneous CGMS System Gold is promising, but real-time glucose level reporting is necessary before it can be of clinical use in the ICU. When implementing a glucose-insulin algorithm in patient care or research, one should realise that the absolute glucose level may differ systematically among various measuring methods, influencing targeted glucose levels.

## Introduction

Critical illness is often accompanied by acute hyperglycaemia, which appears to be a negative prognostic factor for critically ill patients [1-4]. Worse outcome in hyperglycaemic patients has been explained mainly by complications such as infections and decreased wound healing [5]. Moreover, acute hyperglycaemia has a direct effect on the culprit organ(s). A factor that may also contribute to bad prognosis is still the apparent lack of treatment of acute hyperglycaemia.

Recent research suggests that glycaemic control in specific groups of patients reduces morbidity and even mortality. Van den Berghe *et al*. [6] showed that blood glucose regulation toward normoglycaemia decreased mortality and morbidity in a cardiosurgical intensive care unit (ICU) population. Krinsley [7] found a beneficial effect of glucose regulation on mortality in a study using a historical control group. However, in a retrospective study of 1,085 consecutive mixed ICU patients (mortality 20%), using a multivariate analysis model, hyperglycaemia was not an independent factor contributing to mortality [8]. In a recent prospective study in a medical ICU population, van den Berghe *et al*. [9] found that reduced blood glucose levels did not significantly reduce in-hospital mortality for the whole group, only for the subgroup of patients with an ICU stay of 3 days or more. So, the available incidence is inconsistent in regard to the beneficial effects of normoglycaemia. Further progress in establishing the role of glycaemic control in critically ill patients certainly requires an excellent glucose measuring and reporting system. It should give fast and reliable results that can be used in a nurse-driven insulin infusion algorithm that reduces hyperglycaemia without inducing hypoglycaemia [10,11].

A central hospital laboratory delivers glucose values after approximately 30 to 60 minutes, which is too slow to use in a glucose regulation protocol. Literature on point-of-care testing reveals varying accuracy of different handheld meters [12]. The ABL blood gas/glucose analyser used in the study of van den Berghe *et al*. [6], to our knowledge, has not been validated in a critically ill ICU population. Before implementation of a strict glucose protocol in our ICU, we chose to evaluate accuracy and feasibility of the ICU-based blood gas/glucose analyser ABL715; afterward, we tested the reliability of a contemporary handheld point-of-care meter using test strips (Precision PCx; Abbott Diabetes Care, Amersfoort, The Netherlands) and a subcutaneous continuous glucose monitoring system (CGMS) (CGMS System Gold; Medtronic MiniMed, Inc., Northridge, CA, USA).

## Materials and methods

Patients with an expected ICU stay of more than 48 hours were included. Data were collected prospectively. No formal glucose regulation protocol was used; as a rule, continuous insulin infusion therapy was initiated at blood glucose levels exceeding 9 to 10 mmol/l (162 to 180 mg/dl) with a blood glucose target between 6 and 8 mmol/l (at the discretion of the attending physician or nurse). We first validated the ICU-based blood gas/blood glucose analyser ABL715 against the central hospital laboratory (part 1 of the study). Heparinised arterial blood samples were drawn from an arterial line every 6 hours by the same nurse practitioner. One part was sent in a vacuum-sealed plasma separation tube to the central hospital laboratory and analysed by a laboratory technologist using a conventional blood glucose analyser (YSI 2300; Yellow Springs Instruments, Yellow Springs, OH, USA). The YSI analyser uses glucose oxidase to measure the glucose concentration in whole blood (by generation of hydrogen peroxide and gluconic acid from glucose and oxygen via the enzyme glucose oxidase). The other part was immediately analysed on the blood gas/blood glucose analyser ABL715.

Next, part 2 was performed. After inserting (and calibrating) the subcutaneous CGMS System Gold, heparinised arterial blood samples were drawn from an arterial line every 6 hours and analysed by both the Precision PCx point-of-care meter using test strips and the blood gas/glucose analyser ABL715. CGMS glucose data were downloaded after 24 to 72 hours. The continuous glucose monitoring system sensor was inserted in the abdominal subcutis and calibrated every 6 hours according to the manufacturer's indications (using the ABL715 blood glucose values). ICU nurses inspected the insertion site twice daily for signs of infection or bleeding. Data of the CGMS devices were downloaded using the MiniMed Com-Station^® ^and CGMS Solutions™ software (Medtronic MiniMed, Inc.). The institutional medical ethical committee was informed and agreed with the study protocol.

### ABL715

The ABL715 Series (Radiometer Medical ApS, Brønshøj, Denmark) can be set up to measure pH and blood gas and any combination of oximetry, electrolyte, and metabolite parameters. Glucose concentration is measured by the glucose-oxidase method from 95 μl of whole blood to provide results within 2 minutes. Maintenance, calibration, and quality control are performed on a regular basis by the central hospital laboratory. The ABL715 has the capability of cross-linking between the hospital and laboratory information systems, making manual data entry into medical records superfluous. The network connectivity reduces the workload by automating data collection.

### Precision PCx

The Precision PCx point-of-care system (Abbott Diabetes Care) is a portable, glucose oxidase-based, whole-blood testing system using test strips, requiring 3.5 μl of whole blood to provide results within 20 seconds. Results of 4,000 tests can be stored in the monitor itself. The monitor also features a data port and a docking station to automatically download these results in the laboratory or hospital information systems. According to the manufacturer, glucose levels can be measured between 1.1 and 75 mmol/l (20 to 600 mg/dl).

### Continuous Glucose Monitoring System

The CGMS System Gold features a sensor that can be used for up to 72 hours. The sensor consists of a flexible, platinum-plated electrode residing inside a permeable membrane and is inserted in the subcutaneous tissue just under the skin of the abdomen. Subcutaneous glucose is measured by the glucose-oxidase method, and every 10 seconds an interstitial glucose measurement is sent to a monitor that records an average glucose value every 5 minutes. Sensor blood glucose values are calculated using MiniMed Solutions^® ^version 3.0 software. The manufacturer recommends at least four calibrations a day for the CGMS System Gold. Monitor data can be downloaded onto a spreadsheet using the MiniMed Com-Station^® ^and software. The CGMS calculates blood glucose values in the range of 2.2 to 22.2 mmol/l (40 to 400 mg/dl).

### Statistical analysis

Results of paired measurements (laboratory versus ABL715, CGMS versus ABL715, and Precision PCx versus ABL715) were analysed in three ways. Paired values were plotted on a Bland-Altman plot, which is a plot of differences expressed as a percentage of averages between the two methods [13].

The Pearson correlation coefficient (*r*) between the different methods was determined by linear regression. Finally, error-grid analysis was performed to obtain information of clinical relevance from the measurements [14]. Results are plotted in zones of different significance: points in zone A have no clinical implications (clinically accurate measurement), and points in zone B lead to an appropriate clinical decision. Only points lying in zones C, D, and E would lead to inappropriate intervention or lack of intervention. Zone C means misinterpretation of euglycaemia for hyper- or hypoglycaemia (unnecessary overcorrection is possible). Points in zones D and E mean overestimation of hypoglycaemia or underestimation of hyperglycaemia, which may lead to dangerous treatment.

## Results

### Part 1: validation of the blood gas/glucose analyser ABL715

**Figure 1:**
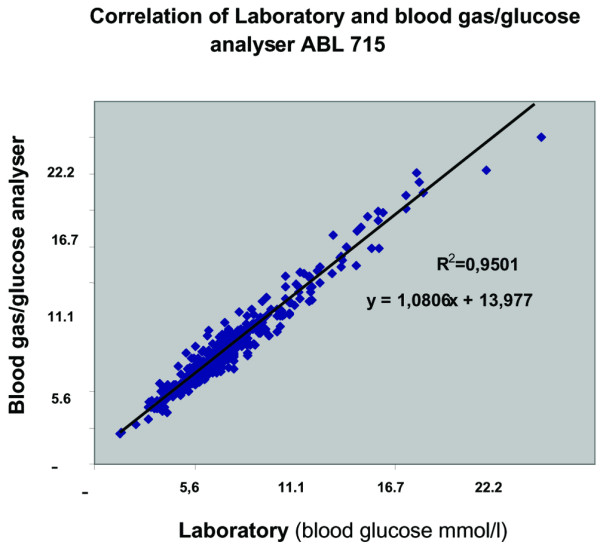
Correlation of laboratory and blood gas/glucose analyser ABL715. Units for blood glucose are millimoles per litre; for conversion to milligrams per decilitre, multiply by 18 (for example, 10 mmol/l = 180 mg/dl.)

**Figure 2:**
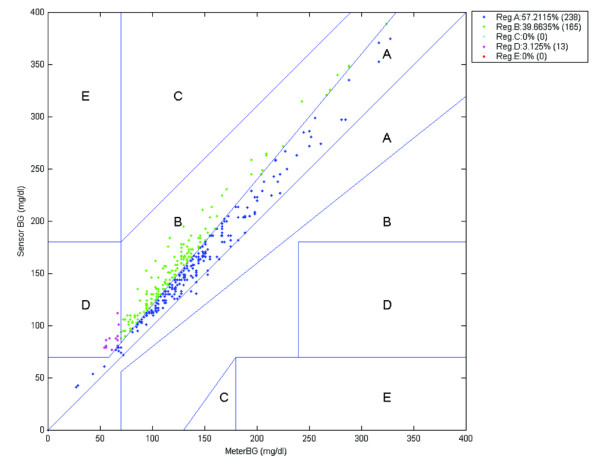
Clarke error grid: laboratory versus blood gas/glucose analyser ABL715. Units are milligrams per decilitre; for conversion to millimoles per litre, divide by 18. BG, blood gas.

Four hundred twenty-four heparinised arterial blood samples were drawn from 45 critically ill ICU patients aged 32 to 88 years. The Pearson correlation coefficient was 0.95 for the ABL715 blood gas/glucose analyser versus laboratory values (Figure 1). In the Clarke error grid, 57.2% of measurements were in zone A and 96.8% in zones A and B (Figure 2). Mean laboratory YSI glucose was 7.5 ± 3.1 mmol/l (135 ± 55.8 mg/dl) versus 8.9 ± 3.4 mmol/l (160.2 ± 61.2 mg/dl) (mean ± standard deviation [SD]) with the ABL715. Over the whole range, the ABL715 displayed blood glucose values that were approximately 18% higher than laboratory values. Only a few glucose levels were in the hypoglycaemic range. The ICU-based blood glucose analyser appeared to be a feasible device that delivers fast and reliable glucose values.

### Part 2: patient characteristics

One hundred sixty-five samples were drawn on 1 to 3 subsequent days per patient from 19 seriously ill ICU patients (aged 31 to 78 years, ICU stay 2 to 25 days [mean 10.7 days]). ICU mortality was 36%. (Our mean ICU mortality is approximately 18%.)

Eighteen patients (of a total of 19 patients) were on mechanical ventilation; three patients needed renal replacement therapy (continuous veno-venous hemofiltration). The admission diagnoses were as follows: pneumonia (3 patients), sepsis (4), chronic obstructive pulmonary disease with pneumonia (2), heart failure (2), aspiration pneumonia (2), HELLP (hemolysis, elevated liver enzymes and low platelet count) syndrome/acute fatty liver (1), gastric bleeding/shock (1), dystrophia myotonica (1), polytrauma (1), and lung transplantation (2).

Values for the mean ± SD (range) of pH, pO_2_, and haematocrit of the glucose blood samples were 7.41 ± 0.08 (7.23 to 7.54), 10.6 ± 2.6 kPa (6.7 to 18.2 kPa), and 0.30 ± 0.06 vol/vol (0.16 to 0.47 vol/vol), respectively.

### Part 2a: validation of the CGMS System Gold

**Figure 3:**
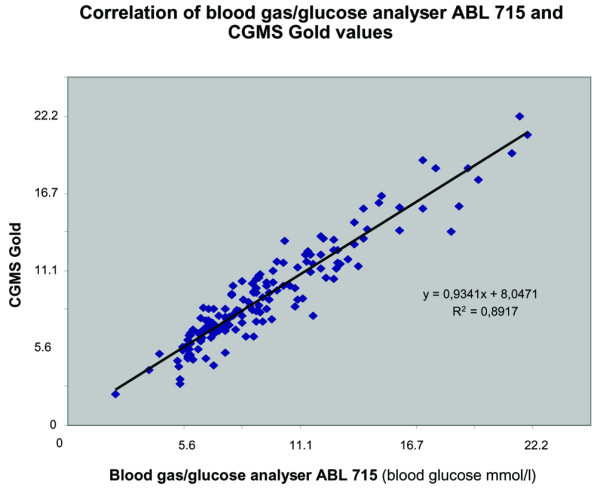
Correlation of blood gas/glucose analyser ABL715 and subcutaneous CGMS System Gold (mmol/l). CGMS, continuous glucose monitoring system.

**Figure 4:**
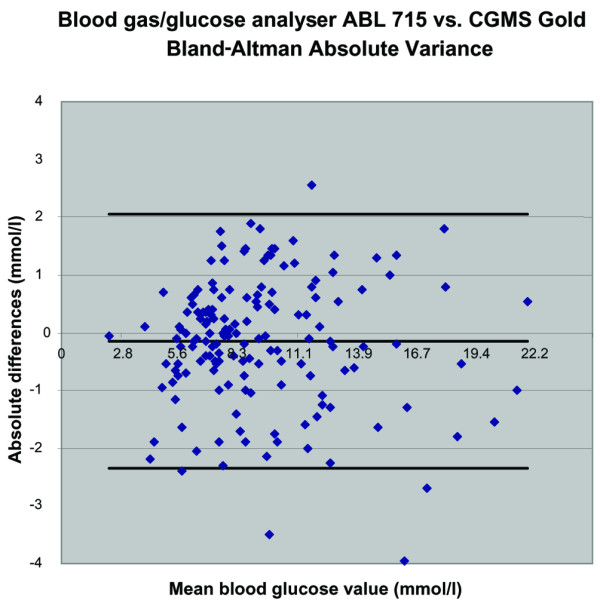
Bland-Altman absolute variance: blood gas/glucose analyser ABL715 versus subcutaneous CGMS System Gold (mmol/l). CGMS, continuous glucose monitoring system.

The Pearson correlation coefficient was 0.89 for CGMS versus blood gas analyser (Figure 3). Ninety-four point three percent of values were inside the 95% confidence interval. In the Clarke error grid, 87.3% were in zone A and 100% were in the clinically acceptable zones A and B. The Bland-Altman plot is shown in Figure 4.

### Part 2b: validation of the Precision PCx point-of-care meter

**Figure 5:**
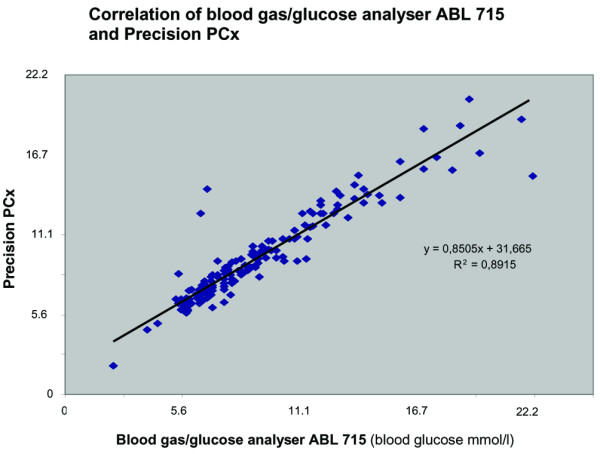
Correlation of blood gas/glucose analyser ABL715 and Precision PCx (mmol/l). CGMS, continuous glucose monitoring system.

**Figure 6:**
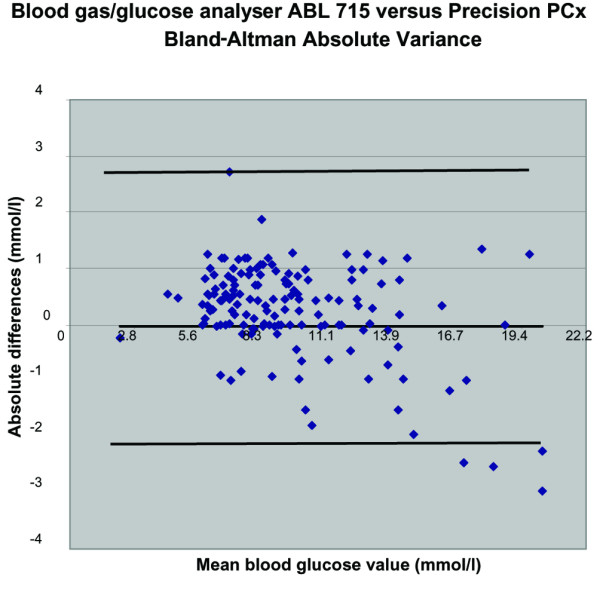
Bland-Altman absolute variance: blood gas/glucose analyser ABL715 versus Precision PCx (mmol/l).

The Pearson correlation coefficient was 0.89 for Precision PCx versus blood gas analyser (Figure 5). Ninety-three point seven percent of measured values were inside the 95% confidence interval. In the Clarke error grid, 95.4% were in zone A and 98.7% were in zones A and B. The Bland-Altman plot is shown in Figure 6.

## Discussion

In this study on seriously ill ICU patients, the blood gas/glucose analyser ABL715 appears to be a good alternative for laboratory testing and can be used for fast and reliable glucose measurements in the ICU. The glucose value is available to the ICU nurse within 2 minutes and is immediately visible in the hospital information system. The ABL715 requires a very small amount of blood; the chance of spilling blood from the syringe or the device is low.

The nursing staff gave the device a high score in terms of user-friendliness and feasibility. As a minor point of criticism, it was mentioned that one ABL715 at a 12-bed ICU occasionally causes a short waiting time. The blood gas/glucose analyser ABL715 systematically gives values approximately 18% higher than the laboratory, although the correlation is very good. This is important when deciding which blood glucose target one aims for in a tight glucose regulation protocol (laboratory value of 6 mmol/l = ABL715 value of approximately 7 mmol/l). When subsequently using this analyser in the ICU, this difference is of minor importance.

The CGMS System Gold also showed a fair correlation. Subcutaneous placing of the sensor's needle was judged to be easy by the research nurse who performed the insertions during this study. The device has to be calibrated four times a day, which requires the availability of another fast and validated blood glucose analyser. This type of CGMS is not yet useful for a glucose regulation protocol, because it provides no real-time glucose data. (They have to be downloaded every 24 to 72 hours.) Soon, newer types will be available which will deliver directly visible 'online' glucose data every 5 minutes.

The Precision PCx point-of-care glucometer showed a fair correlation coefficient, was judged to be user-friendly (in spite of the use of test strips and the higher chance of spilling blood), and delivers fast results.

The turnaround time for glucose determinations by the central hospital laboratory was 30 to 60 minutes, which is too long for fast adjustment of the insulin infusion dose or other therapeutic actions as required in a glucose regulation protocol and might cause excess hypoglycaemia.

Current literature on feasibility of bedside glucometry in critically ill patients is sparse. In a recent hyperinsulinaemic-euglycaemic clamp study in 16 patients with diabetes, Clarke *et al*. [15] found good accuracy for the subcutaneous CGMS in the euglycaemic range: 93.7% of the readings were in error grid zones A and B. In the hypoglycaemic zone (250 blood glucose measurements <3.7 mmol/l [<70 mg/dl]), however, they found the CGMS to be less accurate, with only 62.8% in zones A and B. Goldberg *et al*. [16] also investigated the accuracy of the subcutaneous CGMS System Gold in 21 critically ill patients admitted to a medical ICU and found a Pearson correlation coefficient of 0.88. Clarke error grid analysis categorised 98.7% within the clinically acceptable zones A and B. Only four of more than 500 paired readings were in the hypoglycaemic range in this study [16].

Tang *et al*. [17] tested six handheld glucose meters and a portable glucose analyser. At a glucose concentration of 4.8 mmol/l (86.4 mg/dl) and at a pH between 6.94 and 7.84, glucometry remained accurate. However, at a higher glucose level of 11.2 mmol/l (200 mg/dl), such extreme pH conditions significantly lowered its precision [17]. In another study, bedside reflectance glucometry in 105 arterial blood samples of 10 critically ill adults showed a correlation coefficient of 0.86 compared with the central laboratory [18]. An early report described that fingerstick glucometry was inaccurate among patients in shock [19]. Louie *et al*. [12] compared two point-of-care meters using arterial blood samples obtained from 247 critically ill patients and found that 98% to 100% of the SureStepPro and 91% to 95% of the Precision G measurements fell within an error tolerance of ± 15% versus the hospital chemistry analyser. In a study by Maser *et al*. [20] at a cardiovascular ICU, capillary whole-blood glucometry had a correlation coefficient of 0.88 with arterial plasma samples analysed in the hospital laboratory. In the last study, capillary whole-blood glucose levels were a mean of 0.5 mmol/l (9 mg/dl) lower than arterial plasma specimens.

We found, despite the good correlation coefficient, that glucose values measured by the blood gas/glucose analyser ABL715 were constantly higher than laboratory values and that glucose concentrations measured by the Precision PCx and the CGMS System Gold tended to be lower than the ABL715 values. It is therefore important to realise that defining targets for glucose regulation (for instance, from 4.4 to 6.1 mmol/l [79.2 to 109.8 mg/dl] [6]) also depends on the specific analytic method used at a particular ICU.

It could be questioned which error tolerances are acceptable in a critical care setting. Error tolerance criteria proposed by Kost *et al*. [21] are (a) within ± 0.8 mmol/l (15 mg/dl) of the reference measurement for glucose levels ≤5.6 mmol/l (100 mg/dl) and (b) within approximately 15% of the reference measurement for glucose levels greater than 5.6 mmol/l (100 mg/dl). The ISO (International Organization for Standardization) criteria are as follows: if the reference value is greater than 4.1 mmol/l, the sensor reading should be within 20%; if the reference value is less than 4.1 mmol/l, the sensor reading should be within ± 0.8 mmol/l.

Another way to define the error tolerance and to obtain information of clinical relevance from the measurements is to calculate the Clarke error grid for the glucometer that is evaluated [14]. Defined in this way, all three tested glucometers would be acceptable for use in critically ill patients. We are not completely sure whether this is also true in the hypoglycaemic range, because we did not have many measurements in this range.

Introducing a glucose regulation protocol requires a fast and accurate way to measure blood glucose levels. Because implementing such a protocol would increase nursing workload, it has to be feasible as well. This means that, in all likelihood, not the most accurate, but the most feasible and nevertheless still fairly accurate, glucose analyser would be chosen. On the other hand, the risk of hypoglycaemic episodes could increase. In the sedated, critically ill patient, hypoglycaemic warning symptoms are absent. This means that, apart from safety rules in the protocol, the glucose analyser has to be very reliable in the low range too.

We deliberately choose seriously ill ICU patients, as shown in the patient characteristics, because we aimed to test the reliability of the various analysers under conditions such as shock, vasopressor use, edema, sepsis, and renal replacement therapy. Our study has too few patients and therefore too little data points under extreme conditions of pH, temperature, electrolyte disturbances, and hypoglycaemia to make statements about the reliability of specific analysers under these circumstances.

## Conclusion

The ICU-based blood gas/glucose analyser ABL715 is a rapid and accurate alternative for laboratory glucose determination and can serve as a standard for ICU blood glucose measurements. The Precision PCx is a good alternative, but feasibility is limited because of the blood sample handling. The subcutaneous CGMS System Gold is promising, but real-time glucose level reporting is necessary before it can be of clinical use in the ICU.

When implementing a glucose-insulin algorithm in patient care or research, one should realise that the absolute glucose level may differ systematically among various measuring methods, influencing targeted glucose levels.

## Key messages

• The turnaround time for glucose determinations by the central hospital laboratory is too long for fast adjustment of the insulin infusion dose as required in a glucose regulation protocol.

• The ICU-based blood gas/blood glucose analyser ABL715 is a rapid and accurate alternative for laboratory glucose determination and can serve as a standard for ICU blood glucose measurements.

• When implementing a glucose-insulin algorithm, one should realise that the absolute glucose level may differ systematically among various measuring methods, influencing targeted glucose levels.

## Abbreviations

CGMS = continuous glucose monitoring system; ICU = intensive care unit; SD = standard deviation.

## Competing interests

ICCH serves as a scientific consultant for the continuous glucose measurement research program of Medtronic Europe.

## Authors' contributions

ICCH and JJML initiated and planned the study. AMC, RS, and JJML conducted the study, collected data, and drafted the manuscript. JSWL, JGZ, JET, ICCH, and JHJMM assisted in writing the manuscript. All authors read and approved the final manuscript.
